# Intravenous Vitamin C administration reduces fatigue in office workers: a double-blind randomized controlled trial

**DOI:** 10.1186/1475-2891-11-7

**Published:** 2012-01-20

**Authors:** Sang-Yeon Suh, Woo Kyung Bae, Hong-Yup Ahn, Sung-Eun Choi, Gyou-Chul Jung, Chang Hwan Yeom

**Affiliations:** 1 Department of Family Medicine, Dongguk University Ilsan Hospital, Goyang-si, Gyeonggi-do, Republic of Korea; 2 Department of Medicine, Dongguk University, Seoul, Republic of Korea; 3 Health Promotion Center, Seoul National University Bundang Hospital, Seongnam-si, Gyeonggi-do, Republic of Korea; 4 Department of Statistics, Dongguk University, Seoul, Republic of Korea; 5 Department of Gastroenterology, Asan Medical Center, University of Ulsan, College of Medicine, Seoul, Republic of Korea; 6 Hana Primary Care Clinic, Seoul, Republic of Korea; 7 Yeom Primary Care Clinic, Seoul, Republic of Korea

**Keywords:** vitamin C, office workers, fatigue

## Abstract

**Background:**

Studies of the efficacy of vitamin C treatment for fatigue have yielded inconsistent results. One of the reasons for this inconsistency could be the difference in delivery routes. Therefore, we planned a clinical trial with intravenous vitamin C administration.

**Methods:**

We evaluated the effect of intravenous vitamin C on fatigue in office workers. A group of 141 healthy volunteers, aged 20 to 49 years participated in this randomized, double-blind, controlled clinical trial. The trial group received 10 grams of vitamin C with normal saline intravenously, while the placebo group received normal saline only. Since vitamin C is a well-known antioxidant, oxidative stress was measured. Fatigue score, oxidative stress, and plasma vitamin C levels were measured before intervention, and again two hours and one day after intervention. Adverse events were monitored.

**Results:**

The fatigue scores measured at two hours after intervention and one day after intervention were significantly different between the two groups (p = 0.004); fatigue scores decreased in the vitamin C group after two hours and remained lower for one day. Trial also led to higher plasma vitamin C levels and lower oxidative stress compared to the placebo group (p < 0.001, p < 0.001, respectively). When data analysis was refined by dividing each group into high-baseline and low-baseline subgroups, it was observed that fatigue was reduced in the lower baseline vitamin C level group after two hours and after one day (p = 0.004). The same did not hold for the higher baseline group (p = 0.206).

**Conclusion:**

Thus, intravenous vitamin C reduced fatigue at two hours, and the effect persisted for one day. There were no significant differences in adverse events between two groups. High dose intravenous vitamin C proved to be safe and effective against fatigue in this study.

**Trial Registration:**

The clinical trial registration of this trial is http://ClinicalTrials.govNCT00633581.

## Background

Fatigue is one of the most common complaints in daily life, and the prevalence of fatigue is high in full-time workers. Previous studies have shown that 27% of adults, who were weekly assessed, experienced fatigue [1], and 32.5% of patients who visited primary care clinics complained of fatigue [2]. Oxidative stress is thought to underlie fatigue to some extent; serum markers of oxidative stress are associated with symptoms of chronic fatigue syndrome, including asthenia after physical activity and arthralgias [3]. Such markers of oxidative stress include reactive oxygen species (ROS) and cytokines [4].

Vitamin C is a well-known antioxidant. Several studies have shown that vitamin C can have clinical value in its role as an antioxidant. For example, vitamin C treatment attenuates myalgia and reduces the toxicity of some anticancer agents by reducing oxidative stress [5-8]. Nevertheless, studies of the efficacy of vitamin C supplementation have yielded inconsistent results. Route of administration is one of the major sources of inconsistency. A recent pharmacokinetic study of oral vitamin C showed that plasma vitamin C concentrations are little affected by oral administration, because of poor bioavailability [9]. Since there have been few randomized controlled studies evaluating the efficacy of intravenous vitamin C [10], we sought to reevaluate the effects of intravenous vitamin C. The purpose of this study was to determine the efficacy of high dose vitamin C injection on fatigue in healthy workers, in a randomized controlled trial.

## Materials and methods

### Design

This study was a double-blind, random allocated, placebo-controlled trial conducted at two companies in the Republic of Korea. Participants were recruited between March and April 2008. We randomly assigned 73 participants to the vitamin C group, and 74 participants to the placebo group. The participants received a single intravenous treatment of either vitamin C (10 g) or normal saline. The institutional review board of Dongguk University Ilsan Hospital approved the protocol, and all patients provided written informed consent. The clinical trial registration of this trial is ClinicalTrials.gov NCT00633581.

### Setting and Participants

The study was designed to include 150 volunteers (Korean, office workers or salespeople). The subject size was estimated based on the results of a pilot study with the following parameters: type I error α = 0.05, power 1-β = 0.8, and dropout rate = 10%. The sample variance of the changes in fatigue scores (between pre-trial scores and scores two hours after intervention) was 3.87; the score is described in detail below. The average difference in fatigue score between the vitamin C group and the placebo group was 0.85 (fatigue score) in our pilot study.

The inclusion criteria were as follows: 1) age 20 to 49 years; 2) full-time worker in the company; 3) apparently healthy; 4) no vitamin supplement intake during the two days before enrollment; and 5) voluntary participation. The exclusion criteria were as follows: 1) acute illness (such as common cold or acute gastroenteritis); 2) chronic disease (such as diabetes, hypertension, liver disease, or renal disease); 3) previous history of renal calculi or gout; 4) current pregnancy or lactation; and 5) hypersensitivity to vitamins or intravenous injection. The past medical history was investigated by research nurses and the principal investigator, a board-certified family physician, performed physical examination for the participants.

### Random Assignment and Interventions

We randomly assigned participants to vitamin C and placebo groups. A computerized randomization list was generated by a statistician, sealed in an opaque envelope, and delivered to a study nurse. The study nurse attached consecutive numbers (1-150) to the normal saline bottles, and opened the envelope containing the randomization list in a closed room. She prepared solutions by mixing either vitamin C 10 g (20 ml) or normal saline 20 ml in the normal saline bottle according to the randomization list, then had no further involvement in the study. Another study nurse assigned consecutive numbers (1-150) to the participants in order of enrollment. The solution of the same number was administered to each participant by the study nurses. The participants and study nurses assessing the outcomes were blinded to the group assignment. The vitamin C group received vitamin C 10 g (20 ml of ascorbic acid 500 mg/ml, colorless transparent solution, Merit C^®^, Huons, Korea) in 100 ml of normal saline intravenously over 30 minutes, while the placebo group received the same amount (120 ml) of normal saline in the same manner. The intervention was performed a single time on one day.

### Outcomes and Follow-up

We measured the fatigue score using a numeric rating scale (0-10), with current fatigue (described as "fatigue right now") as a primary outcome [11]. The fatigue score was evaluated three times: before the intervention (baseline), two hours after intervention, and one day after intervention. We determined oxidative stress levels (Free Oxygen Radicals Test (FORT) by Free Oxygen Radicals Monitor Plus, Callegari, Italy) and measured plasma vitamin C concentration (by High Performance Liquid Chromatography, HPLC) at baseline and two hours after the trial. FORT was repeated one day after the trial. The type of detector is UV detector (Hewlett-Packard, German), column used is Cogent(150 mm × 4 mmφ × 5 um)and the buffers are cetyltriethylammonium bromide (SIGMA, U.S.A) and potassium dihydrogen phosphate (DUKSAN, Korea) for the HPLC.

### Assessment of Adverse Events

During intervention and at both post-intervention assessments, study nurses assessed adverse events by asking open-ended questions. Information collected for each adverse event included a description of the event, duration, intensity, required treatment, and outcome.

### Statistical Analysis

Analyses were performed with the "intention-to-treat" method. Predefined primary end-points were the differences in fatigue scores between two hours after intervention, and one day after intervention. The differences in fatigue scores were compared between the vitamin C group and the placebo group using analysis of covariance (ANCOVA), which included baseline fatigue score as a covariate to account for individual variations in fatigue level. As secondary end-points, oxidative stress levels were compared between the two groups using ANCOVA with similar adjustment of baseline levels. Using independent t-tests, plasma vitamin C concentrations were compared between the two groups at baseline and two hours after intervention, respectively. For subgroup analyses, subjects were categorized into two groups (higher and lower) based on the 50^th ^percentile of the baseline vitamin C level. Change in fatigue scores between the trial and placebo group were analyzed using ANCOVA within each group.

Statistical analyses were performed using SPSS, version 16.02 (SPSS, Chicago, IL, USA). Hypothesis tests yielding p values less than 0.05 were considered significant.

## Results

### Subject Compliance to the Clinical Trial

**Figure 1:**
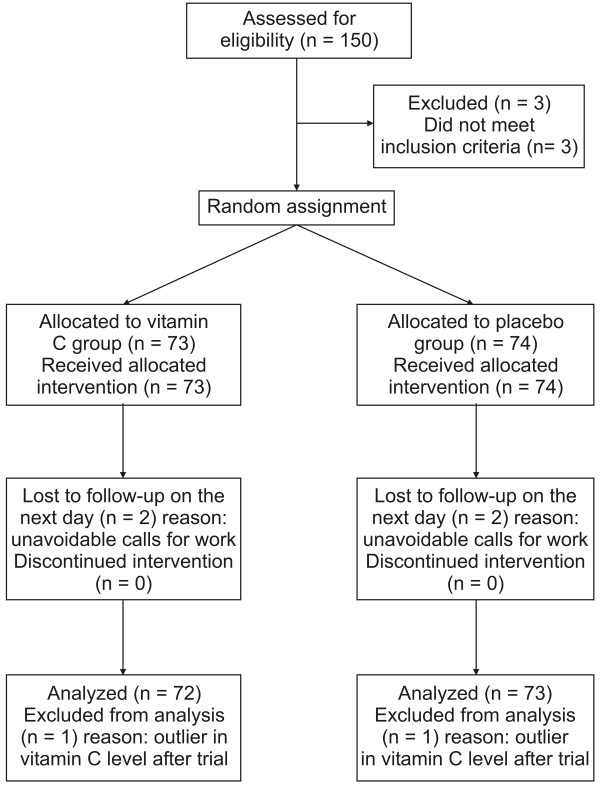
**Study flow diagram**.

Of the 150 subjects initially recruited for the trial, 147 (74 in the vitamin C group, 73 in the placebo group) were enrolled in the final study group. Four participants (two in each group) dropped out of the study the day after intervention. The dropped participants completed two outcome measurements (baseline and two hours after intervention), but did not complete the third measurement (after 24 hour time point) the next day. Thus, we initially analyzed data from the remaining 143 participants. Two outliers (one in each group) for plasma vitamin C level were excluded from analysis. Our final data set, then, included 141 participants (70 in the vitamin C group, 71 in the placebo group) (Figure 1). The baseline characteristics of participants are shown in Table 1. There were no demographical differences between two groups. Lifestyles can influence fatigue; however, it did not differ between the two groups.

**Table 1:** Baseline characteristics of participants.

	Vitamin C Group (n = 70)	Placebo Group (n = 71)	P value
Sex, n			0.559^†^
Male (%)	31 (44.3)	28 (39.4)	
Female (%)	39 (55.7)	43 (60.6)	
Age, years (SD)	30.4 (5.7)	31.2 (5.8)	0.427^‡^
BMI, kg/m^2 ^(SD)	21.5 (3.0)	21.8 (3.1)	0.683^‡^
Usual fatigue, n			0.971^†^
Yes (%)	59 (84.3)	60 (84.5)	
No (%)	11 (15.7)	11 (15.5)	
Smoking status, n			0.158^†^
Smoker (%)	21 (30.0)	14 (19.7)	
Nonsmoker (%)	49 (70.0)	57 (80.3)	
Drinking status, n			0.448^†^
Drinker (%)	56 (80.0)	53 (74.6)	
Nondrinker (%)	14 (20.0)	18 (25.4)	
Exercise, n			0.495^†^
Regular (%)	19 (27.1)	23 (32.4)	
Irregular (%)	51 (72.9)	48 (67.6)	
Sleeping hours (SD)	6.5 (1.0)	6.4 (0.9)	0.296^‡^

†p value from Chi-square test, ‡p value from independent t test, BMI: body mass index

### Impact of Vitamin C on Fatigue

**Figure 2:**
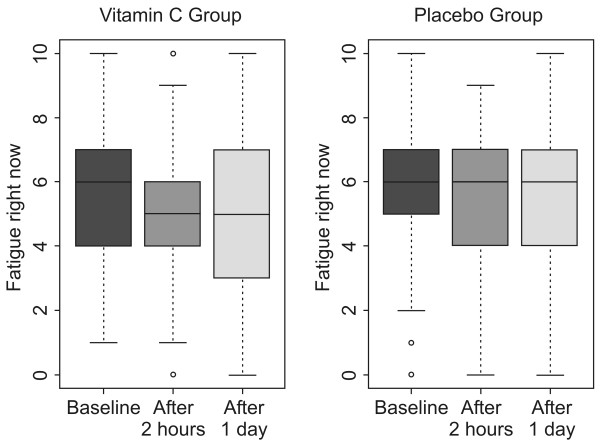
**Boxplots of fatigue scores in the two groups**. The mean fatigue scores of the vitamin C group decreased while those of the placebo group did not.

Fatigue scores (indicating participants' ratings of "fatigue right now") were significantly different between the groups after intervention (p = 0.004, Table 2). There were no significant differences among outcomes assessed two hours after intervention and one day after intervention (data not shown). We conclude that fatigue was significantly improved two hours after vitamin C injection and that the effect persisted one day after the intervention (Figure 2). However, there were no differences between the two groups in "usual fatigue" and "worst fatigue" during the previous 24 hours.

**Table 2:** Comparison of fatigue, plasma vitamin C levels, and oxidative stress between the two groups.

	Vitamin C Group (n = 70)			Placebo Group (n = 71)			p value^‡^
		
	Baseline	2 hours after intervention	1 day after intervention	Baseline	2 hours after intervention	1 day after intervention	
Fatigue right now*	5.64 ± 2.02	5.10 ± 2.04	4.97 ± 2.33	5.54 ± 2.07	5.31 ± 2.00	5.66 ± 2.16	0.004
Usual fatigue during the previous 24 hours	5.59 ± 1.56	-	5.37 ± 2.06	5.77 ± 1.73	-	5.55 ± 1.79	0.870
Worst fatigue during the previous 24 hours	7.16 ± 1.83	-	6.47 ± 2.13	7.14 ± 1.77	-	6.82 ± 1.97	0.202

Plasma vitamin C level (μg/ml)	12.66 ± 6.50	267.90 ± 141.83	-	12.13 ± 4.99	12.52 ± 5.70	-	< 0.001

Oxidative stress (mmol/dl H_2_O_2_)^†^	311.76 ± 74.15	184.46 ± 59.41	296.11 ± 64.37	310.89 ± 74.90	327.21 ± 78.80	303.72 ± 81.26	< 0.001

*Fatigue was measured using a numeric rating scale, 0-10.

†Oxidative stress was measured using the Free Oxygen Radicals Test (FORT).

‡p value from ANCOVA.

### Antioxidant Capacity of Intravenous Vitamin C

Oxidative stress, as the secondary outcome, was significantly lower in the vitamin C group than in the placebo group after intervention (p < 0.001). Plasma vitamin C levels were also significantly different after intervention (p < 0.001).

We have observed in our clinic that some patients with fatigue benefit from vitamin C while others do not; therefore, we tried to find the characteristics of the responsive group. We performed subgroup analysis by categorizing subjects into two subgroups (higher and lower) based on the 50^th ^percentile of the baseline vitamin C level. The 50^th ^percentile of the baseline vitamin C level was 10.97 μg/ml in the trial group and 11.88 μg/ml in the placebo group. We then compared fatigue scores between the trial and placebo groups, in each subgroup. Fatigue score was significantly reduced in the lower group, but not the higher group, compared to placebo (p = 0.004, p = 0.206, respectively, Table 3).

**Table 3:** Comparison of fatigue between the two groups within subgroups categorized by baseline vitamin C level.

		Vitamin C Group (n = 70)			Placebo Group (n = 71)			p value†
			
		Baseline	2 hours after intervention	1 day after intervention	Baseline	2 hours after intervention	1 day after intervention	
Fatigue right now*	Higher	5.74 ± 1.98	5.26 ± 2.23	5.28 ± 2.40	5.47 ± 2.17	5.25 ± 2.30	5.53 ± 2.22	0.206
	Lower	5.54 ± 2.09	4.94 ± 1.85	4.66 ± 2.30	5.60 ± 1.99	5.37 ± 1.68	5.80 ± 2.13	0.004

*Fatigue was measured using a numeric rating scale, 0-10.

Higher and Lower groups were divided by 50th percentile (10.95 μg/ml in trial group, 11.95 μg/ml in placebo group) of baseline plasma vitamin C level.

†p value from ANCOVA with adjustments for the time factor and baseline measurements.

The dropout rate was low (4/147, or 2.7%), and premature withdrawal due to adverse events did not occur in our study. The adverse event profile of the study participants is shown in Table 4. The rate of adverse events in the vitamin C group during and two hours after intervention was 16/70 (22.9%), which was not significantly different from that in the placebo group (11/71, 15.4%). The next day, the rate of adverse events in the vitamin C group was 4/70 (5.7%), which was similar to that in the placebo group (4/71, 5.6%). The most common adverse event in both groups was itching and/or pain at the injection site. The distribution of adverse events did not differ significantly between the two groups at two hours after intervention or the next day (p = 0.266, p = 0.984, respectively). There were no serious adverse events, nor were there adverse events that required treatment.

**Table 4:** Adverse events after intervention.

Characteristics	Vitamin C Group (n = 70)	Placebo Group (n = 71)
Withdrawal from trial for adverse events, n (%)	0 (0)	0 (0)

Patients with ≥ 1 adverse events 2 hours after trial, n (%)	16 (22.9)	11 (15.4)

Patients with ≥ 1 adverse events 1 day after trial, n (%)	4 (5.7)	4 (5.6)

Total adverse events, n	24	17

Patients who experienced specific adverse events 2 hours after intervention, n (%)		
itching sense/pain at injection site	9 (12.9)	8 (11.3)
dry mouth	7 (10)	2 (2.8)
others*	4 (5.7)	3 (4.2)

Patients who experienced specific adverse events 1 day after intervention, n (%)		
itching sense/pain at injection site	1 (1.4)	3 (4.2)
dry mouth	1 (1.4)	0 (0)
diarrhea	1 (1.4)	0 (0)
common cold symptoms	1 (1.4)	1 (1.4)

*Other adverse events included chest discomfort, palpitations, sweating, and abdominal discomfort in the vitamin C group and dizziness, fatigue, and myalgias in the placebo group. These adverse events occurred in one case each.

## Discussion

The primary outcome, fatigue score (assessing the level of "fatigue right now"), was significantly decreased in the vitamin C group compared to the placebo group. The degree of fatigue improved in the vitamin C group 1 day after intervention as well as at 2 hours after intervention. The effect of vitamin C injection was evident in subjects with an initially lower vitamin C level and not in subjects with initially higher levels. Although the pharmacological effect of vitamin C is known to last 4-6 hours (9), our data demonstrate vitamin C injection to be effective a day after intervention as well as at 2 hours after intervention. Previous studies of vitamin C treatment for fatigue have led to disparate results: in some studies, treatment with vitamin C improved fatigue significantly [12,13]; in others, the treatment proved ineffective [14-18]. One trial showed that regular vitamin C infusion reduced fatigue in inpatients with chronic fatigue syndrome [12]. In a Korean study, vitamin C injection (10 g twice a week) improved fatigue in inpatients with terminal cancer [13]. These trials recruited inpatients with serious diseases, and vitamin C administration was done more than two times. However, the studies were all non-comparative trials. In contrast, randomized controlled trials have reported negative results when vitamin C was administered orally [14-18].

Until recently, oral and intravenous vitamin C were regarded as equivalent, but recent data has shown that the two forms have different pharmacokinetic characteristics (9, 10). Plasma concentrations of vitamin C following oral administration are not dose-dependent, and are instead subject to a plateau. Vitamin C distribution after oral delivery is tightly controlled by intestinal absorption, transport to tissues, and renal reabsorption and excretion [19,20]. A recent pharmacokinetic study using the depletion-repletion method showed that intravenous administration could achieve 70-fold higher blood levels of vitamin C compared to the highest tolerated oral dose (9). Intravenous administration of vitamin C bypasses the controls described above, and results in high plasma concentrations. Although we did not use a depletion-repletion study design, we measured plasma levels of vitamin C of 267.90 ± 141.83 μg/ml (mean ± SD) two hours after intervention. This level is equivalent to 1521.04 ± 805.25 μmol/L, overlapping the 1000-1500 μmol/L range predicted from the pharmacokinetic model for 10 g intravenous administration [9].

Our subgroup analysis on the effects on the "fatigue right now" scores indicate that vitamin C injection efficacy is affected by baseline vitamin C levels. Specifically, the subgroup exhibiting an initally lower vitamin C level showed a significant response to vitamin C injection while the subgroup with high baseline levels remained unaffected. We suggest that vitamin C is especially effective in subjects with relatively low baseline levels of vitamin C. Therefore, inconsistent results of previous studies (12, 13, 14-18) may result from route of administration, baseline vitamin C levels of subjects, or both.

Our study has some limitations. First, fatigue measurements were acquired only three times, and plasma vitamin C measurements were made only two times. The association between vitamin C administration and fatigue would have been clearer if we had included more data points in the study. Second, fatigue is a matter of subjective self-assessment. We reduced subjective bias by using a validated measurement tool to assess fatigue [11], and we detected significant differences in fatigue scores after adjustment of baseline values. These findings confirm the likelihood of wide individual variations in fatigue self-assessment. Third, we found no significant relationship between fatigue and oxidative stress. Our participants were healthy adults, so their oxidative stress and fatigue levels might have been too low to demonstrate a clear relationship. Finally, our participants were 20 to 49 years of age and served as office workers and salespeople. Therefore, generalization to labor workers and elderly (≥ 50 years) workers may be limited. Although it is desirable to perform some screening test such as blood chemistry panel, we excluded subjects having chronic disease by past medical history. The reason why we select the dose of vitamin C 10 g is that we considered efficacy and safety for general population. To avoid unexpected adverse reactions, the least dose for the high dose is ideal and we referred a preceding domestic study[13].

We suggest that increase in the dosage or frequency of intravenous vitamin C administration may increase the treatment efficacy. Further study is required to determine effective dose ranges of vitamin C for treatment of fatigue, and serial measurements of fatigue should clarify effective dosage windows.

## Conclusions

We have shown that administration of high dose intravenous vitamin C reduced fatigue significantly compared to placebo in office workers. Moreover, the effect of intervention was strongest in subjects with lower baseline levels of vitamin C and, interestingly, the effect lasted until the next day. These findings have potential clinical implications. Patients with severe fatigue, such as cancer inpatients, and patients at risk for vitamin C deficiency, would exhibit better responses.

## List of abbreviations

ROS: Reactive oxygen species; ANCOVA: Analysis of covariance; FORT: Free Oxygen Radicals Test.

## Competing interests

CHY is the chairperson of the Korean Association for Vitamin Research. GCJ is a member of the Korean Association for Vitamin Research.

## Authors' contributions

SYS developed the study protocol and clinical trial procedures, contributed to the quality control and data cleaning/analysis/interpretation and wrote the first draft of the paper. WKB contributed to the data analysis/interpretation and has been involved in drafting the manuscript. HYA generated the random allocation table, contributed to the quality control and data analysis/interpretation, and has been involved in revising the manuscript critically. SEC helped with data analysis and editing of the paper. CHY contributed the development of the protocol, directed the conduct of the trial, and assisted with drafting the manuscript. GCJ assisted with acquisition of data and editing of the paper. All authors contributed to the design and implementation of the study, reviewed drafts of the manuscript, have read and approved the final draft.

## References

[B1] Bates DW Schmitt W Buchwald D Ware NC Lee J Thoyer E Kornish RJ Komaroff AL Prevalence of fatigue and chronic fatigue syndrome in a primary care practice Arch Intern Med 1993 153 24 2759 2765 10.1001/archinte.1993.00410240067007 8257251

[B2] Skapinakis P Lewis G Meltzer H Clarifying the relationship between unexplained chronic fatigue and psychiatric morbidity: results from a community survey in Great Britain Am J Psychiatry 2000 157 9 1492 1498 10.1176/appi.ajp.157.9.1492 10964867

[B3] Richards RS Roberts TK McGregor NR Dunstan RH Butt HL Blood parameters indicative of oxidative stress are associated with symptom expression in chronic fatigue syndrome Redox Rep 2000 5 1 35 41 10905542 10.1179/rer.2000.5.1.35

[B4] Mantovani G Maccio A Madeddu C Mura L Massa E Gramignano G Lusso MR Murgia V Camboni P Ferreli L Reactive oxygen species, antioxidant mechanisms and serum cytokine levels in cancer patients: impact of an antioxidant treatment J Cell Mol Med 2002 6 4 570 582 10.1111/j.1582-4934.2002.tb00455.x 12611641 PMC6741317

[B5] Bryer SC Goldfarb AH Effect of high dose vitamin C supplementation on muscle soreness, damage, function, and oxidative stress to eccentric exercise Int J Sport Nutr Exerc Metab 2006 16 3 270 280 16948483 10.1123/ijsnem.16.3.270

[B6] Weijl NI Elsendoorn TJ Lentjes EG Hopman GD Wipkink-Bakker A Zwinderman AH Cleton FJ Osanto S Supplementation with antioxidant micronutrients and chemotherapy-induced toxicity in cancer patients treated with cisplatin-based chemotherapy: a randomised, double-blind, placebo-controlled study Eur J Cancer 2004 40 11 1713 1723 10.1016/j.ejca.2004.02.029 15251161

[B7] Sanchez-Moreno C Cano MP de Ancos B Plaza L Olmedilla B Granado F Martin A Consumption of high-pressurized vegetable soup increases plasma vitamin C and decreases oxidative stress and inflammatory biomarkers in healthy humans J Nutr 2004 134 11 3021 3025 15514269 10.1093/jn/134.11.3021

[B8] Cederberg J Siman CM Eriksson UJ Combined treatment with vitamin E and vitamin C decreases oxidative stress and improves fetal outcome in experimental diabetic pregnancy Pediatr Res 2001 49 6 755 762 10.1203/00006450-200106000-00007 11385134

[B9] Padayatty SJ Sun H Wang Y Riordan HD Hewitt SM Katz A Wesley RA Levine M Vitamin C pharmacokinetics: implications for oral and intravenous use Ann Intern Med 2004 140 7 533 537 15068981 10.7326/0003-4819-140-7-200404060-00010

[B10] Padayatty SJ Levine M New insights into the physiology and pharmacology of vitamin C CMAJ 2001 164 3 353 355 11232136 PMC80729

[B11] Yun YH Wang XS Lee JS Roh JW Lee CG Lee WS Lee KS Bang SM Mendoza TR Cleeland CS Validation study of the korean version of the brief fatigue inventory J Pain Symptom Manage 2005 29 2 165 172 10.1016/j.jpainsymman.2004.04.013 15733808

[B12] Kodama M Kodama T The clinical course of interstitial pneumonia alias chronic fatigue syndrome under the control of megadose vitamin C infusion system with dehydroepiandrosterone-cortisol annex Int J Mol Med 2005 15 1 109 116 15583836

[B13] Yeom CH Jung GC Song KJ Changes of terminal cancer patients' health-related quality of life after high dose vitamin C administration J Korean Med Sci 2007 22 1 7 11 10.3346/jkms.2007.22.1.7 17297243 PMC2693571

[B14] Ataka S Tanaka M Nozaki S Mizuma H Mizuno K Tahara T Sugino T Shirai T Kajimoto Y Kuratsune H Effects of Applephenon and ascorbic acid on physical fatigue Nutrition 2007 23 5 419 423 10.1016/j.nut.2007.03.002 17483009

[B15] Close GL Ashton T Cable T Doran D Holloway C McArdle F MacLaren DP Ascorbic acid supplementation does not attenuate post-exercise muscle soreness following muscle-damaging exercise but may delay the recovery process Br J Nutr 2006 95 5 976 981 10.1079/BJN20061732 16611389

[B16] Connolly DA Lauzon C Agnew J Dunn M Reed B The effects of vitamin C supplementation on symptoms of delayed onset muscle soreness J Sports Med Phys Fitness 2006 46 3 462 467 16998453

[B17] Prince MI Mitchison HC Ashley D Burke DA Edwards N Bramble MG James OF Jones DE Oral antioxidant supplementation for fatigue associated with primary biliary cirrhosis: results of a multicentre, randomized, placebo-controlled, cross-over trial Aliment Pharmacol Ther 2003 17 1 137 143 10.1046/j.1365-2036.2003.01398.x 12492743

[B18] Thompson D Williams C McGregor SJ Nicholas CW McArdle F Jackson MJ Powell JR Prolonged vitamin C supplementation and recovery from demanding exercise Int J Sport Nutr Exerc Metab 2001 11 4 466 481 11915781 10.1123/ijsnem.11.4.466

[B19] Levine M Conry-Cantilena C Wang Y Welch RW Washko PW Dhariwal KR Park JB Lazarev A Graumlich JF King J Vitamin C pharmacokinetics in healthy volunteers: evidence for a recommended dietary allowance Proc Natl Acad Sci USA 1996 93 8 3704 3709 10.1073/pnas.93.8.3704 8623000 PMC39676

[B20] Levine M Rumsey SC Daruwala R Park JB Wang Y Criteria and recommendations for vitamin C intake JAMA 1999 281 15 1415 1423 10.1001/jama.281.15.1415 10217058

